# Impact of Kinesiotherapy and Hydrokinetic Therapy on the Rehabilitation of Balance, Gait and Functional Capacity in Patients with Lower Limb Amputation: A Pilot Study

**DOI:** 10.3390/jcm11144108

**Published:** 2022-07-15

**Authors:** Vlad-Theodor Cotrobas-Dascalu, Dana Badau, Marius Stoica, Adina Andreea Dreve, Corina Michaela Lorenta Predescu, Carmen Liliana Gherghel, Mircea Bratu, Popescu Raducu, Antoanela Oltean, Adela Badau

**Affiliations:** 1 Faculty of Physical Education and Sports, National University of Physical Education and Sports, 060057 Bucharest, Romania; cotrobas.vlad@gmail.com (V.-T.C.-D.); adina_dreve@yahoo.com (A.A.D.); corina.predescu@yahoo.com (C.M.L.P.); carmen_gherghel@yahoo.com (C.L.G.); mircea.bratu@yahoo.com (M.B.); 2 Petru Maior Faculty of Sciences and Letters, George Emil Palade University of Medicine, Pharmacy, Sciences and Technology, 540142 Targu Mures, Romania; adela.badau@umfst.ro; 3 Interdisciplinary Doctoral School, Transilvania University of Brasov, 500036 Brasov, Romania; 4 Faculty of Physical Education and Sport, Ovidius University of Constanta, 900470 Constanta, Romania; raducu.popescu22@gmail.com (P.R.); oltean.antoanela@univ-ovidius.ro (A.O.)

**Keywords:** lower limb amputation, rehabilitation, kinesiotherapy, hydrokinetic therapy, balance, gait, functional capacity, pre-prosthetic phase, prosthetic phases

## Abstract

The purpose of this pilot study was to identify impact differences in the rehabilitation of balance, gait and functional capacity in patients with lower limb amputation performing hydrokinetic therapy and kinesiotherapy programs during the pre-prosthetic and prosthetic phases. The study included 16 male patients aged 40–60 years with amputated lower limbs for 6 to 12 months, which involved transfemoral amputation (TFA), transtibial amputation (TTA), traumatic and vascular amputation, who were divided into the following two groups: the hydrokinetic therapy (HKT) group and the kinesiotherapy (KT) group, named after the content of the rehabilitation programs that were implemented for 2 weeks in the pre-prosthetic and prosthetic periods. The initial and final evaluation of the participants included the following tests: the Berg Scale and the four square test for the evaluation of the balance; the PodoSmart device for gait assessment; through the walking test over 6 min, we evaluated the functional capacity. The results were processed in SPSS 24. Analysis of the results on balance rehabilitation through the Berg Scale highlighted that the progress related to the mean of the total score was 7.62 points, *p* = 0.00 for the HKT group and 7.50 points, *p* = 0.00 for the KT group, while in the four square step test, the mean of progress was 6.125 s, *p* = 0.00 for the HKT group and 6 s, *p* = 0.000 for the KT group. The PodoSmart gait analysis revealed that the HKT group showed a progress mean of 4.875%, *p* = 0.00, for the foot symmetry parameter, which was 1.875% less than the score achieved by the KT group whose symmetry progress mean was 6.75%, *p* = 0.00, while the average progress mean for the cadence parameter was 2.75 steps/min higher for the KT group than the HKT group. The comparative analysis of the impact of these two programs on the patients’ functional capacity indicated that the score recorded by the KT group was a progress mean of 15.12 m, *p* = 0.00 better than the HKT group for the travelled distance parameter; the implementation of the hydrokinetic therapy program led to better exercise adaptation for the HKT group compared to the KT group at an average HR (HRavg) with 0.50 BPM, *p* = 0.00. After analyzing the results, it has been found that hydrokinetic therapy programs have a greater impact on balance rehabilitation and exercise adaptation, while kinesiotherapy programs have a greater impact on gait rehabilitation and functional capacity optimization for the travelled distance parameter.

## 1. Introduction

Amputation refers to the surgical removal of a limb, part of a limb or part of the body. The major causes of lower extremity amputation are complex and varied, including chronic vascular disease, diabetes and trauma, which are followed by the onset of a very high rate of disability and handicap of the musculoskeletal system. The frequency of amputations of different etiologies has significantly increased over the last 25 years, and this number is expected to double in the next 15 years [1,2,3,4]. The prosthetic possibilities have been continuously improved, relying on the principles of gait biomechanics. Lower limb amputation is a permanent surgical procedure with major functional, psychological and social sequelae that are likely to affect the quality of life [5,6,7].

A major challenge for physiotherapists in the case of partial or total amputation of the lower limb is the optimization of motor and functional capabilities, with a focus on the rehabilitation of patients’ balance and gait [8,9]. Studies reveal that lower limb amputation leads to an imbalance in the biomechanics and biostatics of the whole body in general, but especially in the amputated extremity [10,11]. The lack of a segment of the lower limb causes motor deficit in the muscle and joint groups, with disruption of functional capabilities due to imbalanced gait patterns [12,13]. The absence of the amputated segment induces partial and global activity restrictions. The resulting dysfunctions lead to the onset of disability, which has an influence on the patient’s psycho-emotional status, independence, quality of life and life expectancy [14,15,16].

In Romania, the total number of amputations is constantly growing; it has been concluded that the incidence of lower limb amputations is a quality marker of diabetic foot management, and a high rate of amputations can be associated with poor patient education, late presentation to the doctor and insufficient resources. In the last decade, the range of motor activities aimed at increasing the quality of life has diversified; thus, one can choose according to their preferences, material living conditions, etc. [17,18,19,20,21]. Studies show that approximately 90% of amputation cases involve the lower limbs, and the incidence of these amputations in Europe has increased considerably in the last 25 years, ranging from 180–200 patients to 1 million and it is estimated that this negative dynamic will continue in the coming 15–20 years [22,23,24,25,26].

The motor and functional rehabilitation of patients with lower limb amputation is mainly achieved through various physical therapy exercises [27,28,29,30]. Over the past decade, kinetic rehabilitation programs have been considerably diversified as a result of developing modern motor and functional recovery devices and equipment and adapting information technologies to the needs of patients with lower limb amputation [31,32]. Kinesiotherapy includes various physical exercises, balance, walking and functional training in a comprehensive general plan of care. Kinesiotherapy helps to improve adaptation to new static and dynamic conditions, improve the postural balance, walking ability and climbing/descending stairs, regaining gait capacity. The patient can perform the exercises without the supervision of the therapist at home. A disadvantage of physical therapy is that incorrect exercise can affect the abutment and its injury. This trend also includes the adaptation of kinesiotherapy to the aquatic environment, which is called hydrokinetic therapy and aims at rehabilitating patients with lower limb conditions and amputations, due to the multiple benefits of practicing in the water environment and taking advantage of materials, equipment and devices designed to be used in immersion [33,34,35].

Hydrokinetic therapy refers to partial immersion movement therapy, which is based on exercising with the entire body or only some of its parts, while benefiting from the properties and characteristics of the aquatic environment. Hydrokinetic therapy has many positive effects on improving health, neuromotor and functional rehabilitation potential and mental abilities [36,37]. Balance, muscle strength and proprioception can be better addressed in the aquatic environment, especially during adulthood and old age [38,39]. Exercising in the aquatic environment enhances the nervous excitation and muscle relaxation processes, which creates conditions for the patients’ vestibular disinhibition and in-water practice [40,41]. Hydrokinetic therapy promotes venous circulation, which facilitates the reduction in edema in the abutment, decreases joint overload and pain symptoms, increases the threshold of pain sensitivity, stimulating the thermoregulation and the metabolism, sensory stimulation, muscle relaxation, creating exercises with progressive resistance, re-education of balance and subjective feelings of well-being. Studies highlight that water recovery exercise is indicated for patients with musculoskeletal impairments, and the effectiveness of these programs depends on the time of starting kinetic rehabilitation, the length of implementation, the materials and equipment used and the exercise content complexity [42,43]. As for the disadvantages of hydrokinetic therapy, we can observe that it is expensive; special equipment is required; it must be performed under specialized supervision; the water temperature must be at 30°; there is a short duration of sessions (30–45 min); one cannot regain gait with prosthesis; patients with chlorine allergy, heart problems or water phobia cannot participate.

According to the laws of physics, the gravitational force and implicitly the joint and muscle loading decrease concomitantly with the immersion depth, and this allows diversifying global and segmental movement possibilities, regaining muscle tone and accelerating clinical-functional and motor rehabilitation [44,45]. Researchers have found that water immersion to the sacral area reduces body weight by over 50%, thus lowering pressure and stress on the lower extremities [46,47,48]. The characteristics of the aquatic environment in swimming pools have the potential to facilitate blood circulation to the joints involved in movement, relax muscles and temporarily reduce pain. Due to the viscosity and density properties of water, people with impaired balance who exercise in the aquatic environment can slow down their imbalance speed and correct their postural and movement errors that may cause falls and injuries [49,50,51,52].

There is relatively little research that aims to identify the impact of hydrokinetic therapy programs compared to kinesiotherapy programs on the motor and clinical-functional rehabilitation of patients with lower limb amputation. Based on the aforementioned arguments, the purpose of this pilot study was to identify impact differences in the rehabilitation of balance, gait and functional capacity in patients with lower limb amputation performing hydrokinetic therapy and kinesiotherapy programs during the pre-prosthetic and prosthetic phases.

The research hypothesis assumes that the rehabilitation of motor and clinical-functional capabilities, more specifically balance, gait and functional capacity in patients with lower limb amputation, will have a different impact on patients performing hydrokinetic therapy programs compared to those performing physical therapy programs in the pre-prosthetic and prosthetic periods.

## 2. Materials and Methods

### 2.1. Participants

This pilot study included 16 patients in the pre-prosthetic and prosthetic phases due to workplace amputation injuries. They were divided into two equal groups in accordance with the agreement to practice the hydrokinetic or kinesiotherapy programs as follows: the experimental group that performed the hydrokinetic therapy program and the control group that performed the kinesiotherapy program. Inclusion criteria were as follows: transfemoral amputation (TFA), transtibial amputation (TTA), age between 40 and 60 years, amputated lower limb for 6 to 12 months, traumatic and vascular amputation, male gender. Exclusion criteria were as follows: amputation level other than thigh and lower leg, bilateral amputation, age under 40 years and over 60 years, severe mental health problems, hypertension, sphincter incontinence, respiratory failure, fear of drowning and chlorine allergy.

### 2.2. Study Design

The pilot study was conducted at the National Institute of Medical Expertise and Work Capacity Recovery in Bucharest, which has a physiotherapy room and a hydrokinetic therapy pool designed for the rehabilitation of patients with amputation. The study was structured in the following stages: for KT, an initial test (It) from 8 to 10 September 2021, implementation of the physical therapy program between 11 September and 12 November 2021 and a final test (Ft) from 13 to 15 October 2021; for HKT, an initial test (It) from 18 to 20 November 2021, implementation of the hydrokinetic therapy program between 21 November 2021 and 22 January 2022 and a final test (Ft) from 23 to 25 January 2022.

The kinetic protocols for KT and HKT were applied to patients in the pre-prosthetic and prosthetic phases, during which the following objectives were pursued: stump modelling and maturation, increasing muscle strength and endurance, maintaining and increasing joint mobility, re-educating balance and coordination, re-educating prosthetic gait, cardiovascular and respiratory training and developing programs for activities of daily living.

The kinetic rehabilitation sessions for HKT and KT groups were performed 5 times a week over a 2-week period (during hospitalization) and lasted about 30–45 min each program. The two programs were similar in terms of session duration and implementation period, but both physical therapy and hydrokinetic programs were constantly adjusted to the changing clinical condition of the patients in terms of particularizing the dosage of the exercises, reducing or increasing the complexity of the exercises and the variation in the exercise intensity. The kinetotherapy program included posture and sitting and standing exercises, exercises with the amputated foot in the main direction of muscle toning, exercises in the stand to improve balance, walking exercises with or without support, ascending and descending exercises, etc. The hydrokinetic therapeutic program included exercises for lifting, lowering, pedaling, bending, extensions in immersion with support at the edge of the pool or on the wand floating exercises, swimming exercises, walking exercises, etc. The hydrokinetic therapy program took place in a pool with a water depth of 0.5–1.6 m, which allowed most patients to be in water to the chest level. The water temperature was 30 °C (±3 °C). The kinesiotherapy program took place in the rehabilitation room inside the hospital. 

The study was conducted in accordance with the principles set out in the Declaration of Helsinki. Written informed consent was obtained from all participants. The study was approved by the Ethics Commission of the National University of Physical Education and Sport in Bucharest, with no. 373/11.04.2022. 

### 2.3. Assessment

To investigate the impact of rehabilitation programs on patients with lower limb amputation, our study aimed at assessing balance and gait. Balance was assessed using the Berg Scale and the four square step test, gait was assessed with the PodoSmart device, and functional capacity was assessed by the 6-min walk test (6 MWT). The tests were applied one day before the implementation of the individual rehabilitation programs and at the end of it. The initial and final tests were applied under similar conditions for all study subjects, in the same recovery room, at the same time and in the same order. The Berg Scale is used to measure balance and the risk of falling during functional tasks [53]. This scale includes the following tasks to be performed by patients: sitting unsupported, standing to sitting, sitting to standing, transfers, standing unsupported, standing with eyes closed, standing with feet together, standing with one foot in front, standing on one foot, turning to look behind, retrieving object from floor, turning 360 degrees, pacing alternate foot on stool, reaching forward with outstretched arm. The 14 tasks based on functional activities are scored from 0 to 4, where 0—unable to perform, 1—needs assistance to prevent falling, 2—able to perform with great difficulty, 3—able to perform with minimal difficulty, 4—performs without difficulty. The Berg Scale scoring can range from 0 points, which is associated with a severely impaired balance diagnosis, up to 56 points, which means excellent functional balance.

The four square step test assesses dynamic balance by a person’s ability to step over obstacles in several directions, including forward, sideways, backward. The test involves the rapid transfer of body weight from one foot to the other during changes in direction, as well as stepping over low objects. Normal value is considered a score of <24 s, which indicates no risk of falling, while a score of >24 s is associated with impaired balance and risk of falling.

The PodoSmart gait analysis uses PodoSmart equipment [54], which is designed for healthcare professionals to improve the clinical assessment of patients with various mobility disorders, but we used it in our study to assess gait. PodoSmart insoles consist of wireless sensors that can be fitted into any shoe and offer the possibility to measure spatial, temporal and kinematic gait parameters. The PodoSmart insole measurement frequency is 2400–2483.5 MHz, with a maximum power of 10 mW. The maximum body pressure supported is 50 kg/cm^2^, and the maximum load of the insoles is 200 kg [54].

PodoSmart insoles consist of wireless sensors that can be fitted into any shoe and offer the possibility to measure spatial, temporal and kinematic gait parameters. The intelligent insoles have several sensors that detect and capture foot movements and a microprocessor that calculates gait-related biomechanical data [55]. PedoSmart sensors attached to insoles are completely wireless, are pressure sensors with integrated internal storage and have the ability to analyze in real time 20 gait variables [55,56,57]. The test consists of a 6–8-min walk, during which in-depth information is collected about the patient’s walking profile in terms of cadence and symmetry in real life conditions. Cadence (or stride rate) is the number of steps per minute (as the sum of both feet).

Symmetry expresses the congruence between the values obtained for the right and left foot, making it possible to determine whether a foot is used more than the other during locomotion; the main parameter taken into consideration is the duration of contact unilateral podal with the ground.

The 6-min walk test facilitates the assessment of exercise capacity using the Polar H10 chest strap heart rate monitor [58]. The test consists of identifying minimum, maximum and average heart rates as well as the distance a patient can cover on a flat surface within 6 min. This test usually expresses a submaximal level of exercise capacity (the maximum normal value for healthy people is 700 m), with most patients reaching their own level of exercise rather than the maximum normal level [59].

### 2.4. Statistical Analysis

For this study, the data were processed using SPSS 24, and the statistical parameters used were as follows: mean (X), mean difference between tests (∆XFt-It), U—Mann–Whitney U test, Z—Z test, r—effect size, median. For spread distribution we used IQR—interquartile range, Q1-Q3—quartile first and third (25–75%). Interpretation of the Cohen’s (r) effect size is as follows: 0.1–0.2—small, 0.3–0.5—medium, 0.5–0.8—large, over 0.8—very large. For this study, a *p*-value of < 0.05 was chosen as reference for statistical significance. Symmetry expresses the congruence between the values obtained for the right and left foot, making it possible to determine whether a foot is used more than the other during locomotion; the main parameter taken into consideration is the duration of contact unilateral podal with the ground. 

## 3. Results

In the tables in this section, we highlighted the most relevant results on the characteristics of the samples, the results of the tests to assess the balance, gait and functional capacity of patients with lower limb amputation in the pre- and post-prosthetic period.

According to Table 1, it can be observed that the differences in the arithmetic means between the two groups were 1.03 years, 0.50 cm height, 0.78 kg weight and 1.07 IMC. The distribution of patients according to the type of amputation and the level of amputation are shown in Table 1. Four age parameters for the groups of the study results of the Mann–Whitney U tests were as follows: U = 29.50, HKT no = 8, KT no = 8, Z = 0.26, *p* = 0.792; for height, we obtained the results U = 31, HKT no = 8, KT no = 8, Z = 0.105, *p* = 0.916; for weight, U = 30.50, HKT no = 8, KT no = 8, Z = 0.15, *p* = 0.874; and for IMC, the results were U = 20.50, HKT no = 8, KT no = 8, Z = 0.24, *p* = 0.532. There were no statistically significant differences between the study groups in any of the parameters of age, weight, height and BMI (Table 1).

Table 2 shows the results obtained by the HKT and KT groups in the 14 specific tasks of the Berg Scale. The result analysis reveals that all arithmetic means for all components fall between the upper and lower limits of the 95% confidence interval for both groups participating in the pilot study.

The mean difference between the initial and final tests for HKT and KT was identical in the following Berg Scale tasks: sitting unsupported—0.12 points; transfers—0.12 points; standing unsupported—0.62 points; standing with eyes closed—0 points; turning 360 degrees—0.75 points; reaching forward with outstretched arm—0.75 points. By analyzing the differences in arithmetic means, it is found that the progress made by the HKT group is higher compared to the KT group in the following tasks: standing with feet together—0.12 points; standing with one foot in—0.12 points; placing alternate foot on stool—0.25 points; instead, the KT group recorded larger mean differences than the HKT group in the following tasks: standing to sitting—0.02 points; sitting to standing—0.25 points; standing on one foot—0.125 points; retrieving object from floor—0.12 points.

Analysis of the Berg Scale total score (Table 3) showed that the progress was 7.62 points for the HKT group, while the KT group progressed by only 7.50 points between tests. According to the IQR of the Berg Scale total score of the hydrokinetic therapy group, the middle 50% of values in the dataset had a spread of 16.75 for the initial test and 14 for the final test, respectively; for the kinesiotherapy group, 13.75 for the initial test and 9.50 for the final test. The results of the initial tests between the groups HKT and KT were insignificant statistics for all parameters according to the Mann–Whitney test. For the initial test, the Scale Berg total score between the HKT and KT groups revealed that U = 12, HKT no = 8, KT no = 8, Z = 2.11 and *p* = 0.235 (statistically significant), with a large effect size r = 0.53; for the final test, compared with the KT with HKT group, the results were U = 15, HKT no = 8, KT no = 8, Z = 1.81 and *p* = 0.069 (insignificant statistics), with r = 0.45, showing a medium effect size.

According to Table 3, for the four square step test for the assessment of dynamic balance in HKT and KT groups, the results were statistically significant, with the *p* values recorded in the study being lower than the selected significance threshold of *p* < 0.05. For the four square step test, the results of the Mann–Whitney U test for the initial tests, by comparing the KT group with HKT group, highlight that U = 9.50, HKT no = 8, KT no = 8, Z = 2.37, *p* = 0.118, with a large effect size r = 0.53; and for the final test, the results were U = 6, HKT no = 8, KT no = 8, Z = 2.75, *p* = 0.006 and r = 0.68, a value that also highlights a large effect size. After implementing the experimental pilot programs, the HKT group progressed by 6.12 points, and KT by 6 points, with the difference between samples being 0.12 points for the HKT group. The IQR of the hydrokinetic therapy group and the middle 50% of values in the dataset had a spread of 6.25 for the initial test and 3.75 for the final test, respectively; for the kinesiotherapy group, 6.75 for the initial test and 3.25 for the final test.

In the PodoSmart gait analysis, the HKT group progressed between tests (after implementing the hydrokinetic therapy program) by 4.87% for the foot symmetry parameter, which was 1.87% less than in the KT group, whose symmetry progress between tests was 6.75%. Cadence analysis showed that the average progress for the KT group was higher by 2.75 steps per minute compared to the HKT group (Table 4). For foot symmetry, the IQR of the hydrokinetic therapy group and the middle 50% of values in the dataset had a spread of 81.25 for the initial test and 89.5 for the final test, respectively; for the kinesiotherapy group 79.25, for the initial test and 87.25 for the final test. For cadence, the second parameters of PodoSmart, the IQR of the hydrokinetic therapy group revealed a spread of 79.5 for the initial test and 98.25 for the final test, respectively; for kinesiotherapy group, 83.5 for the initial test and 91.5 for the final test (Table 4).

The results of the initial tests between the groups KT and HKT were insignificant statistics for all parameters according to the Mann–Whitney test. For the initial test of the foot symmetry, the results of the Mann–Whitney U test were as follows: for HKT group, U = 14, HKT no = 8, KT no = 8, Z = 1.90, *p* = 0.365, with a medium effect size r = 0.47; and for the final test, the results were U = 11.50, HKT no = 8, KT no = 8, Z = 2.16, *p* = 0.028 and r = 0.68, a value that highlights a large effect size; for the initial test for the cadence, the results were U = 16, HKT no = 8, KT no = 8, Z = 1.68, *p* = 0.105, with a medium effect size r = 0.42; and for final test, the results were U = 14.50, HKT no = 8, KT no = 8, Z = 1.84, *p* = 0.045 and r = 0.46, a value that also highlights a medium effect size.

Results for the six MWT functional capacity assessments reveal that the mean differences between tests and groups are statistically significant regarding the following parameters: travelled distance, and average heart rate (HRavg), while for minimum and maximum heart rate (HR min; HRmax), the *p*-values are higher than the reference *p*-value of <0.05. The results of the initial tests between the groups of study (HKT and KT) were insignificant statistics for all parameters according to the Mann–Whitney Test.

We detailed the final results for the Mann–Whitney U test regarding only the distance traveled and HRavg parameters due to the confirmed statistical significance. For the travelled distance, the results of the initial test between the HKT and KT groups were U = 22, HKT no = 8, KT no = 8, Z = 1.45, *p* = 0.092, with a medium effect size r = 0.36; and for final test group, the results were U = 22.50, HKT no = 8, KT no =8, Z = 1.09, *p* = 0.006 and r = 0.27, a value that highlights a small effect size; for the HRavg, the results of the initial test were U = 32, HKT no = 8, KT no = 8, Z = 2.76, *p* = 0.064, r = 0.69, which revealed a large effect size; and for the final test between the HKT and KT groups, the results were U = 15.50, HKT no = 8, KT no = 8, Z = 1.74, *p* = 0.041 and r = 0.43, a value that highlights a medium effect size.

For travelled distance, the IQR of the hydrokinetic therapy group and the middle 50% of values in the dataset had a spread of 142.50 for the initial test and 124.50 for final test, respectively; for the kinesiotherapy group, 175 for the initial test and 168.25 for the final test. For HRavg, the IQR of the hydrokinetic therapy group and the middle 50% of values in the dataset had a spread of 8 for the initial test and 9.50 for the final test, respectively; for the kinesiotherapy group, 12 for the initial test and 14.75 for the final test. The comparative analysis of the mean differences recorded by the KT group compared to the HKT group shows that the score obtained by the KT group is 15.12 m better than that of the HKT group only for the travelled distance parameter; regarding the other HR parameters, the mean differences recorded between the two tests emphasize that the implementation of the hydrokinetic therapy program has led to better exercise adaptation for the HKT group compared to the KT group as follows: at a minimum HR, with 1.75 beats per minute (BPM); at a maximum HR, with 0.50 BPM; at an average HR, with 0.50 BPM (Table 5).

## 4. Discussion

This study aimed to identify impact differences in the rehabilitation of balance, gait and functional capacity in patients with lower limb amputation performing hydrokinetic therapy and kinesiotherapy programs during the pre-prosthetic and prosthetic phases.

We believe that the research results contribute to expanding the therapists’ knowledge about the role and impact of kinesiotherapy programs, but especially hydrokinetic therapy programs on the motor and clinical-functional rehabilitation of patients with lower limb amputation. The analysis of the pilot study results highlights that there are statistically significant differences between the final and initial tests for the assessment of balance, gait and functional capacity. The design, adaptation and implementation of a hydrokinetic therapy program for patients with lower limb amputation in the pre-prosthetic and prosthetic phases are some novelty aspects that, according to the results of our study, will support the optimization and rehabilitation of balance and exercise adaptation, which will be reflected in heart rate variations, due to the properties of the aquatic environment and the multiple practice possibilities. The research results also suggest a greater impact of kinesiotherapy programs on gait and functional capacity rehabilitation regarding the travelled distance parameter in patients with lower limb amputation, compared to patients performing the hydrokinetic therapy program. These results are also confirmed by previous studies that highlight the role of kinetic exercise in muscle toning and the rehabilitation of gait biomechanics and dynamics in patients with lower limb amputation [60,61,62]. Research on this category of patients has mainly focused on issues related to pain therapy [63,64], prosthesis accommodation [65,66], improving motor and functional capabilities [67,68,69], psychological impact, increasing quality of life [70,71] and social and professional integration [72,73]. The clinical-functional rehabilitation of patients with lower limb amputation requires the concentrated and competent activity of orthopedic surgeons, physiotherapists, psychiatrists, psychotherapists, internal medicine specialists and social workers [74,75]. Studies show that the earlier the implementation of specialized exercise programs, the more effective the possibilities for motor and functional rehabilitation [60,75]. Numerous studies have shown that exercise adapted to different pathologies contributes greatly to the rehabilitation of motor and functional functions, and their effectiveness is dependent on the period of exercise and the specificity of the exercises [76,77,78,79]. The interdisciplinary medical approach [80,81,82,83,84], focused in an integrative and complex way, influences the prophylactic, interventional and post-interventional activity of patients with amputation [85,86,87]. According to the results of our study, recent research demonstrates the positive role of the use of hydrokinesiotherapy on the quality of life of patients with various pathologies specific to the musculoskeletal system, respiratory and cardiovascular system, mental state, depression, etc. whose incidence increased during the COVID-19 pandemic [88,89,90].

The strengths of this study were as follows: the study aimed at designing and implementing two exercise-based interventions, more specifically, a kinesiotherapy program and an innovative hydrokinetic therapy program for the clinical-functional and motor rehabilitation of patients with lower limb amputation, in order to identify the impact of these programs on the improvement of balance, gait and functional capacity. For this purpose, we persuaded eight patients to attend the hydrokinetic therapy program by accepting immersion in the pool water to perform three categories of tests for the assessment of their gait, balance and functional capacity.

The limitations of this study were as follows: involvement of a relatively small number of patients; involvement of only male patients because our attempts to persuade female patients were unsuccessful; the lack of a true control group, a relatively limited period to conduct exercise programs for patients with lower limb amputation; lack of analysis and other proprioceptive and motor parameters.

## 5. Conclusions

The results of our study contribute to expanding the understanding of the impact that the implementation of hydrokinetotherapy and kinetotherapy programs has on the process of motor and functional rehabilitation on patients with lower limb amputation in the pre-prosthetic and prosthetic period. The analysis of the results of patients with lower limb amputations included in the research samples facilitates the understanding that rehabilitation programs must be individualized regarding the content, dosage, intensity and complexity of the exercise. Based on the results of our study, we can conclude that both programs have contributed to optimizing the process of motor and functional rehabilitation, and we believe that the combination of different types of kinetic programs adapted to the terrestrial environment (indoor, indoor) or aquatic (swimming pool, indoor) will have a major impact on rehabilitating the balance, gait and functional capacity of patients with lower limb amputation in the pre-prosthetic and prosthetic period.

Balance rehabilitation and HR adaptation to exercise are more effective when implementing hydrokinetic therapy programs due to the water properties and temperature, as well as the various possibilities of action and movement of the body and segments in the aquatic environment. Kinesiotherapy programs had a greater impact on gait rehabilitation and functional capacity optimization with regard to the travelled distance parameter compared to hydrokinetic therapy programs. We believe that diversifying the contents of recovery exercise programs and applying them as early as possible can play a major role in the motor and functional rehabilitation of patients with lower limb amputation in the pre-prosthetic and prosthetic phases. Furthermore, the adaptation of kinetic programs to the aquatic environment can optimize the rehabilitation process of certain proprioceptive, motor and functional components of the amputated limb, and thus reduce the degree of disability. The combination of physiotherapy and hydrokinetotherapy programs can help streamline the rehabilitation of patients with lower limb amputation in terms of recovery time and effects.

## Figures and Tables

**Table 1:** Statistical analysis and distribution of characteristic of study groups.

Group	Parameters	Age	Height (cm)	Weight (kg)	IMC	Period Amputation (Month)	TFA (No.)	TTA (No.)	Traumatic Amputation (No.)	Vascular Amputation (No.)
HKT	X	51.75	178.87	84.37	25.37	8.6	3	5	6	2
	Median	53	179	85	25.68	-	-	-	-	-
	IQR (Q1; Q3)	12 (45.75; 57.75)	6.5 (175.25; 181.75)	8.75 (79.75; 85)	10.25 (24.25; 27.75)	-	-	-	-	-
KT	X	52.77	179.37	85.11	26.45	8.4	4	4	5	3
	Median	51.50	179	86.50	26.29	-	-	-	-	-
	IQR (Q1; Q3)	11.25 (45.25; 56.5)	15.50 (170.25; 185.75)	13.25 (76.25; 89.5)	11.25 (24.5; 27.5)	-	-	-	-	-

HKT—hydrokinetic therapy, KT—kinesiotherapy, X—arithmetic mean, IMC—body mass index, TFA—transfemoral amputation, TTA—transtibial amputation, IQR—interquartile range, Q1–Q3—quartile 1–3 (25–75%).

**Table 2:** Statistical analysis of Berg Scale task results.

Parameters	Group	It-X	Ft-X	ΔX Ft-It	Median-It	Median-It	ΔMedian Ft-It
Sitting unsupported	HKT	3.875	4.000	0.125	4	4	-
	KT	3.875	4.000	0.125	4	4	-
Standing to sitting	HKT	1.875	3.000	1.125	1.5	3	1.5
	KT	2.000	3.250	1.250	2	3	1
Sitting to standing	HKT	2.750	3.125	0.375	3	3	-
	KT	2.625	3.125	0.500	3	3	-
Transfers	HKT	3.000	3.125	0.125	3	3.5	0.5
	KT	3.125	3.250	0.125	3	3	-
Standing unsupported	HKT	2.875	3.500	0.625	3	4	1
	KT	3.000	3.625	0.625	4	4	-
Standing with eyes closed	HKT	3.750	3.750	0.000	4	4	-
	KT	3.750	3.750	0.000	4	4	-
Standing with feet together	HKT	3.500	3.750	0.250	3	4	1
	KT	3.625	3.750	0.125	4	3	1
Standing with one foot in front	HKT	3.125	3.750	0.625	3	4	1
	KT	3.250	3.750	0.500	3	4	1
Standing on one foot	HKT	.625	1.125	0.500	0.5	1	0.5
	KT	.625	1.125	0.625	0.5	1.5	1
Turning to look behind	HKT	3.625	4.000	0.375	4	4	
	KT	3.625	3.875	0.250	4	4	-
Retrieving object from floor	HKT	2.875	3.500	0.625	3	3.5	0.5
	KT	2.750	3.500	0.750	3	3.5	0.5
Turning 360 degrees	HKT	2.125	2.875	0.750	2	3	1
	KT	3.250	3.000	0.750	3	3	-
Placing alternate foot on stool	HKT	0.875	2.250	1.375	0.5	2.5	2
	KT	1.500	2.625	1.125	2	3	1
Reaching forward with outstretched arm	HKT	3.000	3.750	0.750	3	4	1
	KT	3.000	3.750	0.750	3	4	1

HKT—hydrokinetic therapy, KT—kinesiotherapy, It—initial test, Ft—final test, X—arithmetic mean, ∆X Ft-It—mean difference between tests, ∆Median Ft-It—median difference between tests.

**Table 3:** Statistical analysis of the Berg Scale total score and four square step test results.

Test	Group	It-X	It-Median	It-IQR (Q1; Q3)	Ft-X	Ft-Median	Ft-IQR (Q1; Q3)	ΔX Ft-It
Berg Scale (points)	HKT	37.87	37.50	16.75 (29; 25.45)	45.50	48	14 (37; 51)	10.24
	KT	39	46.50	13.75 (32.25; 46)	46.50	48	9.50 (41.50; 51)	7.50
Four Square Step Test (s)	HKT	27.62	28.50	6.25 (23.75; 30)	21.50	21.50	3.75 (20; 23.75)	6.12
	KT	28.25	28.50	6.75 (24; 30.75)	22.25	22	3.25 (20; 23.25)	6

HKT—hydrokinetic therapy, KT—kinesiotherapy, It—initial test, Ft—final test, X—arithmetic mean, ∆X Ft-It—mean difference between tests, IQR—interquartile range, Q1–Q3—quartile 1–3 (25–75%).

**Table 4:** Statistical analysis of PodoSmart test results.

Test	Group	It-X	It-Median	It-IQR (Q1; Q3)	Ft-X	Ft-Median	Ft-IQR (Q1; Q3)	ΔX Ft-It
Foot symmetry (%)	HKT	85.75	85	8.25 (81.25; 89.50)	90.62	89	7 (87.25; 94.25)	4.87
	KT	82.87	81.50	8 (79.25; 87.25)	89.62	89	6.75 (87; 93.75)	6.75
Cadence (number of steps per minute)	HKT	89.25	86	14.75 (83.50; 98.25)	96.00	95	13.50 (90; 103.50)	6.75
	KT	86.12	87	8 (83.50; 91.50)	95.62	99	14.50 (89; 103.50)	9.50

HKT—hydrokinetic therapy, KT—kinesiotherapy, It—initial test, Ft—final test, X—arithmetic mean, ∆X Ft-It—mean difference between tests, 9 IQR—interquartile range, Q1–Q3—quartile 1–3 (25–75%).

**Table 5:** Statistical analysis of 6-min walk test results.

Test	Group	It-X	It-Median	It-IQR (Q1; Q3)	Ft-X	It-Median	It-IQR (Q1; Q3)	ΔX Ft-It
Travelled distance (m)	HKT	268.75	250	142.50 (195.50; 338)	301.12	295	124.50 (230.50; 355)	32.37
	KT	230.50	197	175 (159.75; 334.75)	278	258.50	168.25 (201.75; 370)	47.50
HRmin (BPM)	HKT	85.50	85	9.25 (80.75; 90)	96.87	96	10.50 (92.75; 103.25)	11.37
	KT	85	86	8.25 (81.25; 89.50)	98.12	98.50	16 (91.25; 107.25)	13.12
HRmax (BPM)	HKT	129.50	129.50	14 (122.25; 136.25)	133.37	133.50	4.50 (130.50; 135)	3.87
	KT	128.75	128	11.50 (121.50; 133)	133.12	133.50	5.25 (129.75; 135)	4.37
HRavg (BPM)	HKT	112.12	112.50	8 (108.50; 116.50)	118.37	118	9.5 (115; 124.50)	6.25
	KT	111.87	113	12 (105.25; 117.25)	117.75	117.50	4.75 (115; 119.75)	5.87

HKT—hydrokinetic therapy, KT—kinesiotherapy, It—initial test, Ft—final test, X—arithmetic mean, ∆X Ft-It—mean difference between tests, IQR—interquartile range, Q1–Q3—quartile 1–3 (25–75%).
